# CXCL12/SDF-1 and CXCR4

**DOI:** 10.3389/fimmu.2015.00301

**Published:** 2015-06-12

**Authors:** Takashi Nagasawa

**Affiliations:** ^1^Department of Immunobiology and Hematology, Institute for Frontier Medical Sciences, Kyoto University, Kyoto, Japan; ^2^Core Research for Evolutional Science and Technology (CREST), Japan Science and Technology Agency (JST), Kyoto, Japan

**Keywords:** CXCL12, CXCR4, bone marrow, niche, stem cells, hematopoietic stem cells, B cell, Foxc1

Chemokines are a large family of structurally related chemoattractive cytokines, which have four conserved cysteines forming two disulfide bonds, and act through seven-transmembrane-spanning receptors coupled to heterotrimeric GTP-binding proteins (G-protein-coupled receptors). Chemokines were thought to be signaling molecules that attract leukocytes to sites of inflammation; however, CXC chemokine ligand (CXCL)12 [also known as stromal cell-derived factor (SDF)-1α and pre-B-cell-growth-stimulating factor (PBSF)] is the first member that was shown to be critical for developmental processes, including hematopoiesis (1), cardiogenesis (1–3), vascular formation (2), and neurogenesis (3), as well as the maintenance of tissue stem cells (4).

## Identification of CXCL12

Our interest is how bone marrow microenvironments regulate hematopoiesis, including B lymphopoiesis. To address this, we tried to identify a cytokine, which was important for B cell development in the marrow. In 1988, Namen et al. identified interleukin 7 (IL-7) produced by a bone marrow-derived stromal cell line as a cytokine, which enhanced the proliferation of B cell precursors. However, several studies suggested that IL-7 was not sufficient to support B lymphopoiesis. Hayashi et al. speculated that at first stage in B cell development, progenitors depended on unidentified molecules produced by the stromal cell line called PA6 alone for proliferation and differentiation into the second stage, where progenitors depended on both PA6-derived factors and IL-7 for proliferation (5).

It was unclear whether PA6-derived factors were soluble factors or not in Hayashi’s model (5). To address this issue, we cultured bone marrow hematopoietic cells in the absence or presence of PA6 cells separated by a membrane filter, allowing the passage of proteins but not cells. We showed that while very few viable B cell precursors were present 7 days after the culture of bone marrow hematopoietic cells in the presence of IL-7 and absence of PA6 cells, the proliferation of B cell precursors were enhanced in the presence of PA6 and IL-7. These findings suggested the existence of soluble factors produced by PA6 cells that stimulated the proliferation of B cell precursors in the presence of IL-7 (6).

We tried to develop more simple culture system suitable for molecular cloning and found that a stromal cell-dependent B cell precursor clone, DW34, which was established from Whitlock-Witte-type culture by limiting dilution on a stromal cell line, could proliferate in the presence of a conditioned medium from PA6 cells (6). An expression cDNA library was prepared from PA6 cells using the vector pME18S, and then more than 10^4^ pools were screened for the activity to stimulate the growth of DW34 cells after enforced expression in COS-7 cells, and positive pool was subdivided until a single positive clone was identified. We revealed that a conditioned medium from the positive clone-transfected COS-7 cells had DW34 growth stimulating activity and termed this molecule PBSF (6). The nucleotide sequence and deduced amino acid sequence of the clone were determined and its product was identical to a chemokine called SDF-1α (6, 7). We felt these results somewhat disappointing because chemokines were thought to be rather inflammatory mediators at that time. In 1993, Tashiro et al. developed a method for molecular cloning of cDNAs that contain signal sequences, such as those encoding secreted proteins and receptors without the use of specific functional assays, and identified SDF-1α; however, its function was unclear (7). Thus, we revealed that SDF-1α/PBSF (now formally named CXCL12) stimulated the proliferation of B cell precursors (6).

## Identification of a Receptor for CXCL12

All known chemokine receptors are G-protein-coupled receptors (GPCR) and amino acid sequence is conserved among these molecules. Based on this, we synthesized four degenerate oligonucleotides corresponding to conserved amino acid sequences in transmembrane regions of the chemokine receptors, including murine CXCR2, CCR2, and human HUMSTR, and used them as primers in PCR experiments to identify chemokine receptors abundantly expressed by murine CXCL12 responsive DW34 cells (8). The deduced amino acid sequence of a cDNA yielded by this approach shared 90% amino acid identity with previously identified human HUMSTR/HM89/LESTR/fusin, a HIV-1 entry co-receptor and designated murine HUMSTR/HM89/LESTR/fusin (now formally named CXCR4) (8). CXCL12 induced an increase in intracellular free Ca^2+^ in DW34 cells and CXCR4-transfected Chinese hamster ovary (CHO) cells, suggesting that CXCR4 is a receptor for CXCL12 (8). On the other hand, Bleul et al. and Oberlin et al. demonstrated that human HUMSTR/HM89/LESTR/fusin is a receptor for human CXCL12 (9, 10). The majority of chemokine receptors recognize more than one chemokine, and many chemokines bind to more than one chemokine receptor. However, we and others revealed that mice lacking CXCR4 showed hematopoietic and cardiovascular phenotypes strikingly similar to those of CXCL12 deficient mice, as described below, indicating that CXCR4 is the primary physiologic receptor for CXCL12 in mammals (1–3).

## Essential Physiological Roles of CXCL12–CXCR4 Signaling

To determine the role of CXCL12 in hematopoiesis, we generated and analyzed CXCL12 and CXCR4 deficient mice, which died perinatally. Consistent with the activities of CXCL12 in promoting the proliferation of B cell precursors (6), CXCL12-CXCR4 signaling was essential for the development of B cells from the earliest precursors in fetal liver and bone marrow (1, 11). Surprisingly, CXCL12-CXCR4 signaling was also essential for homing of hematopoietic stem cells (HSCs) and neutrophils to fetal bone marrow during ontogeny (1–3, 12). Subsequently, we generated CXCR4 conditionally deficient mice and revealed that CXCL12-CXCR4 signaling was essential for the maintenance of HSCs, the production of immune cells, including B cells, plasmacytoid dendritic cells (pDCs), which expressed high levels of type I interferon (IFN), and were thought to play important roles in antiviral immunity, and NK cells and homing of end-stage B cells, plasma cells into bone marrow (4, 11, 13). In addition to hematopoiesis, we found that CXCL12-CXCR4 signaling was essential for homing of primordial germ cells (PGCs) to gonads, a cardiac ventricular septal formation and vascularization of the gastrointestinal tract during ontogeny (1–3). In the meantime, Littmann’s group described that CXCR4 was essential for migration of granule cells in appropriate positions in the cerebellum during neurogenesis (3), and besides these additional physiological roles of CXCL12-CXCR4 signaling, other groups revealed its relevant pathological roles. In 1996, Feng et al. found that CXCR4 acted as an essential co-receptor for T cell-tropic strains of human immunodeficiency virus type-1 (HIV-1), and Bleul et al. and Oberlin et al. demonstrated that CXCL12 had HIV-suppressive activities (9, 10). Furthermore, CXCL12-CXCR4 signaling has been reported to be involved in migration of cancer cells, including presumptive cancer stem cells, to sites of metastasis and increased their survival and/or growth in various cancers, such as breast and lung cancers, as well as leukemia and lymphoma.

## CXCL12-Expressing Cells in Bone Marrow

**Figure 1:**
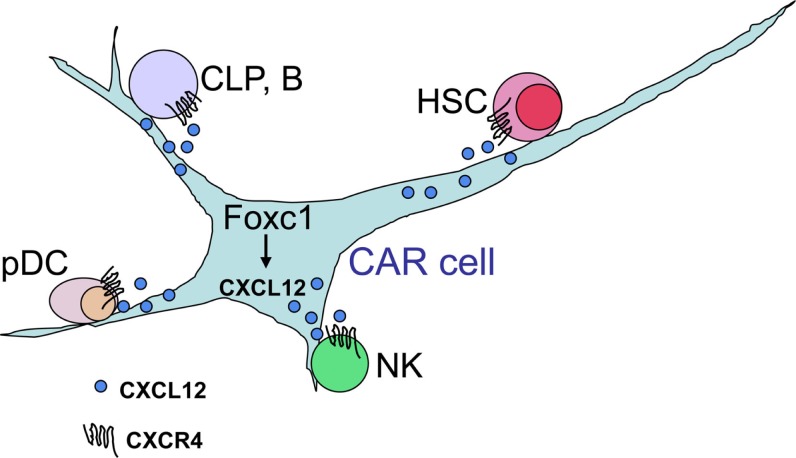
**CXCL12-abundant reticular (CAR) cells**. In adult bone marrow, the transcription factor Foxc1 induces development of CAR cells and maintains bone marrow niches for HSPCs, up-regulating the expression of CXCL12, which is essential for the maintenance of HSCs, common lymphoid progenitors (CLPs), B cells, pDCs, and NK cells, in CAR cells.

As the CXCL12-CXCR4 signaling plays a key role in hematopoiesis, we were prompted to visualize cells, which expressed CXCL12 in bone marrow. For this, we generated mice with the green fluorescent protein (GFP) reporter gene knocked into the CXCL12 locus and found that CXCL12 as well as stem cell factor (SCF), which was essential for HSC proliferation, was preferentially expressed in a population of stromal cells with long processes, termed CXCL12-abundant reticular (CAR) cells (11–13). CAR cells are adipo-osteogenic progenitors, which express adipogenic and osteogenic genes, including peroxisome proliferator-activated receptor γ (PPARγ) and Osterix (Osx), and largely overlap with SCF-expressing cells predominantly expressing leptin receptor (Lepr) (13–15). Histological analysis showed that most HSCs and very early B cell progenitors were in contact with CAR cells (4, 11), and the experiments using diphtheria toxin-based system that allows the inducible, short-term ablation of CAR cells *in vivo* revealed that CAR cells were essential for maintenance of hematopoietic stem and progenitor cells (HSPCs) in bone marrow (Figure 1) (14). Recently, we found that the transcription factor Foxc1 was expressed preferentially in CAR cells and was essential for CAR cell development and maintenance of bone marrow niches for HSPCs up-regulating SCF and CXCL12, which plays major roles in HSC maintenance and immune cell production (Figure 1) (15).

Taken together, CXCL12 and CXCR4 have been identified as key spatiotemporal regulators of migratory stem and progenitor cell behavior, and our studies provide considerable new insights into the biology and pathology of tissue stem cells as well as hematopoiesis, vasculogenesis, and neurogenesis, and in some cases, for clinical application in various diseases.

## Conflict of Interest Statement

The author declares that the research was conducted in the absence of any commercial or financial relationships that could be construed as a potential conflict of interest.
